# DTI Study on Brain Structure and Cognitive Function in Patients with Chronic Mountain Sickness

**DOI:** 10.1038/s41598-019-55498-9

**Published:** 2019-12-18

**Authors:** Haihua Bao, Ruiyang Li, Mingli He, Dongjie Kang, Lili Zhao

**Affiliations:** Department of Medical Imaging Center, Affiliated Hospital of Qinghai University, Xining, Qinghai China

**Keywords:** Neurological disorders, Outcomes research

## Abstract

In chronic mountain sickness (CMS) patients, the structure of the brain, memory and cognition are often irreversibly damaged by chronic hypoxia due to red blood cell overcompensation, elevated haemoglobin and blood stasis. In this study, we aimed to evaluate this damage using diffusion tensor imaging (DTI) and to study the correlations among the fractional anisotropy (FA),the apparent diffusion coefficient (ADC) value, the severity index of CMS and the simple Mental State Examination (MMSE) score in CMS patients. A total of 17 patients with CMS and 15 healthy controls were recruited for conventional brain magnetic resonance imaging (MRI) and DTI scans, and ADC images were reconstructed along with FA and FA colour maps. The FA and ADC values of the selected regions of interest (ROIs) were measured and compared. The FA and ADC values were also compared with the haemoglobin (Hb) and MMSE scores. CMS patients are prone to intracranial ischaemia, infarction and haemorrhage. Multiple structural changes occur in the brain of CMS patients, and these changes are related to the severity of the disease and cognitive function variation. The white matter fibre bundles of CMS patients showed no obvious damage, except in the ischaemic site.

## Introduction

Chronic mountain sickness (CMS) is a common and frequently occurring disease that threatens the population living on the high-altitude plateau. CMS can cause damage to multiple organs, especially the brain and heart^1^. Brain damage, such as cerebral edema, and neurological deterioration could be related to the structural changes of the white matter fibre bundle, microstructural changes within the gray matter, and cognitive function changes. These changes can potentially be studied using the magnetic resonance imaging (MRI) diffusion method called diffusion tensor imaging (DTI), which can characterize the diffusion anisotropy for the human brain.

DTI, through calibrating the movement direction of water molecules within the tissue, can clearly visualise the anisotropic feature of white matter fiber^2^. DTI is currently the only imaging method that can non-invasivly display internal fibre structures in the brain^3^. With DTI, clinicians and researchers can directly see the orientation and connection of white matter fiber bundles of human brain^4^.and DTI has been widely used in preoperative evaluation, pathological research and other fields.

The fractional anisotropy (FA) value reflects the proportion of the water molecule anisotropy component in the whole diffusion tensor and indirectly reflects the integrity of white matter fibre bundles. The main function of the apparent diffusion coefficient (ADC) value is to quantitatively analyse the dispersion degree of molecules, and this value represents the diffusion efficiency of water molecules in tissues^2^. Mini-mental state examination (MMSE)^5^ is the most widely used simple cognitive function assessment scale,This questionnaire can reflect the intelligence status and the degree of cognitive impairment in the subjects comprehensively, accurately and rapidly.

Mukherjee *et al*.^6^ found that DTI showed more ischaemic changes in the white matter of the brain than diffusion-weighted imaging (DWI). Foong^7^ used DTI technology to study patients with schizophrenia. This study suggests a local disruption of the fibrous junction of the compression of the corpus callosum in schizophrenia patients. Christopher^8^ used the DTI technique to study intracranial lesions in patients with HIV. The reduction in the CD4 T cell count in HIV patients was correlated with diffusion anisotropy. The study also found that subfrontal cortex white matter was most likely to be involved in HIV infections.

In this study, DTI was used to measure the FA and ADC values in different regions of the brains in patients with CMS. In addition, an MMSE was conducted. The aim is to determine whether any correlations exist among CMS severity measured by haemoglobin content (Hb), brain cognitive function measured by MMSE, and brain tissue diffusion anisotropy measured by FA in CMS patients.

## Results

(1) In the CMS group, 11 patients had sulcus, shallow fission and narrowing of the ventricular system. In total, 15 patients (including patients with intracranial haemorrhage) had multiple incidents of cerebral ischaemia and intracranial infarction, and 3 patients had intracranial infarction and haemorrhage. (2) Compared with the control group, in the CMS group, the FA value of the right frontal white matter area decreased (p < 0.05), and the ADC value of the right internal capsule forelimb increased (p < 0.05). The FA value of the caudate nucleus on the left side and the ADC value of the left thalamus were positively correlated with Hb (r = 0.53, p = 0.03; r = 0.67, p < 0.05). The FA value of the left internal capsule and the ADC value of the right hippocampus were negatively correlated with the MMSE score (r = −0.67, p < 0.05; r = −0.590, p < 0.05). (3) In the CMS group, some of the white matter fibre bundles in the patients with intracranial haemorrhage showed partial rupture and distortion when passing through the ischaemic site.

**Figure 1:**
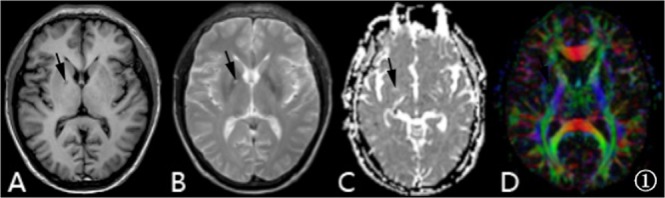
A 42-year-old male patient with CMS, Hb 220 g/l. Images (**A–D**) are T1-weighted, T2-weighted, ADC and FA colour maps, respectively. The black arrow points to the intracranial haemorrhage foci in the right basal ganglia region.

Routine brain MRI, FA and ADC were acquired for all subjects. In the CMS group, there were 11 patients with cerebral sulci, superficial fission, narrowing ventricular system, 15 patients (including the haemorrhage patients) with multiple ischaemic foci and infarction in the skull, and 3 patients with complicated intracranial haemorrhage foci, as shown in Fig. 1, which displays the images of one CMS patient. In both the control and CMS groups, the FA and ADC values from the frontal white matter, corpus callosum, internal capsule, and caudate nucleus were measured and compared. The right frontal lobe white matter FA value in the CMS group was lower than that in the control group (t = 2.74, p < 0.05) (see Table 1); the ADC values of the right side of the internal capsule forelimb in the CMS group were higher than those in the control group (t = 2.35, p < 0.05) (see Table 2).

**Table 1:** Comparison of FA values between the CMS group and the control group.

Groups	N	White matter of the frontal lobe		Anterior limb of the internal capsule	
		Left side	Right side	Left side	Right side
CMS group	17	0.341 ± 0.069	0.345 ± 0.077	0.649 ± 0.151	0.671 ± 0.098
Control group	15	0.370 ± 0.096	0.417 ± 0.070	0.624 ± 0.129	0.667 ± 0.089
t value		1.00	2.74	0.404	2.353
p value		0.33	0.01	0.625	0.894

**Table 2:** Comparison of ADC (×10^−3^ mm^2^/s) between the CMS group and the control group.

Groups	N	White matter of the frontal lobe		Anterior limb of the internal capsule	
		Left side	Right side	Left side	Right side
CMS group	17	0.811 ± 0.057	0.835 ± 0.075	0.701 ± 0.129	0.816 ± 0.119
Control group	15	0.792 ± 0.045	0.822 ± 0.050	0.682 ± 0.143	0.717 ± 0.119
t value		0.99	0.58	0.40	2.35
p value		0.33	0.57	0.69	0.03

**Figure 2:**
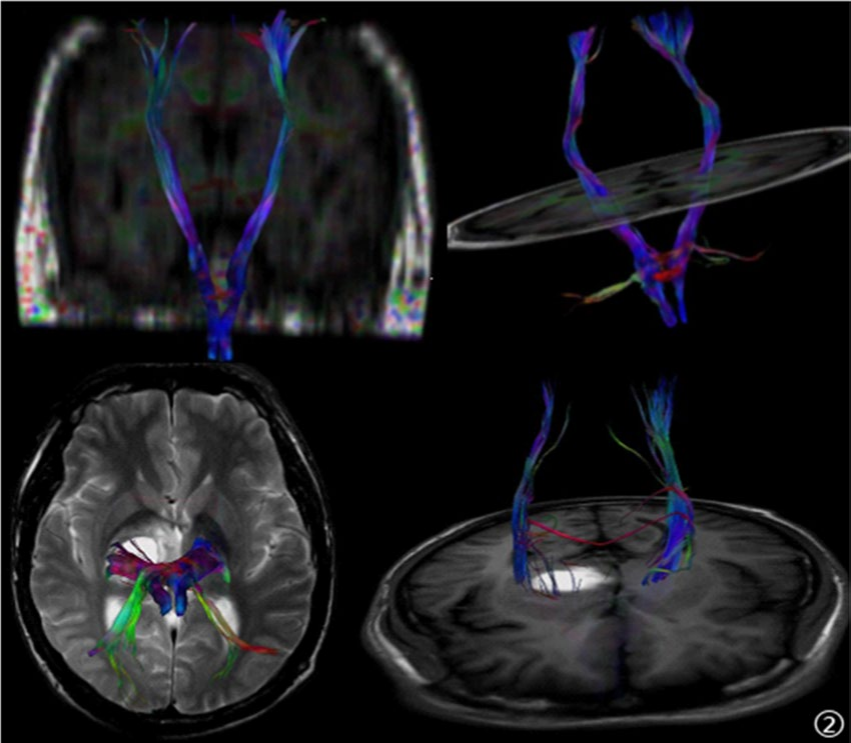
A 41-year-old male patient with CMS, headaches and dizziness. The right thalamus and basal ganglia had haemorrhagic foci. The right cortical spinal tract is misshapen, showing a possible rupture.

In the CMS group, the FA value of left caudate nucleus and ADC value of left thalamus were positively correlated with the Hb value, while the FA value of left anterior capsule and ADC value of right hippocampus were negatively correlated with MMSE score (see Tables 3 and 4). Fibre bundle imaging showed no obvious abnormalities in the white matter fibre bundle of the two groups. The fibre bundle was sparse and fractured in the haemorrhage site of 3 patients with intracranial haemorrhage in the CMS group (see Fig. 2)

**Table 3:** Correlation between the FA value and haemoglobin/MMSE score based on the ROIs of the patients with CMS.

N			Anterior limb of the				Hippocampus			
			internal capsule		Caudate nucleus		Cerebral ganglia		japonicas	
			left side right side		left side right side		left side right side		left side right side	
Hb	17	r value	0.275	0.342	0.533	−0.237	−0.359	0.023	−0.215	−0.273
		P value	0.286	0.178	0.027	0.360	0.157	0.929	0.406	0.290
MMSE	14	r value	−0.667	−0.337	−0.008	0.070	0.528	0.079	−0.139	0.203
		P value	0.009	0.239	0.978	0.813	0.052	0.790	0.635	0.485

**Table 4:** Correlation between ADC (×10^−3^ mm^2^/s) and Hb (g/L)/MMSE scores based on the ROIs of patients with CMS.

N			Anterior limb of the				Hippocampus			
			internal capsule		Caudate nucleus		Cerebral ganglia		japonicas	
			left side right side		left side right side		left side right side		left side right side	
HGB	17	r value	−0.121	−0.204	−0.478	0.278	0.674	0.144	−0.032	−0.111
		P value	0.643	0.432	0.052	0.281	0.003	0.581	0.904	0.672
MMSE	14	r value	0.225	−0.111	−0.349	−0.177	−0.323	−0.129	0.246	−0.590
		P value	0.439	0.706	0.222	0.546	0.260	0.660	0.397	0.026

## Discussion

Chronic mountain disease is a common clinical syndrome in immigrants and native residents who live for years at an altitude of more than 2,500 metres. The main manifestation of chronic mountain disease is elevated erythrocytes (female Hb is greater than 190 g/L and male Hb is greater than 210 g/L^9^) due to the gradual loss of acclimatization in low-oxygen environments of the plateau. Studies by Hackett *et al*.^10^ have shown that under high-altitude conditions, such as low temperature, low pressure and low partial pressure of oxygen, the pituitary adrenal medulla is hyperactive, and a large number of vasoactive substances, such as catecholamine, enter into the blood circulation. At the same time, aldosterone and antidiuretic hormone levels become elevated, resulting in water and sodium retention and erythrocytosis. An excessive elevation in erythrocytes will result in high blood viscosity and blood flow resistance, slow and deposition of blood flow, and reduced blood flow in tissues^11^. Patients with chronic mountain disease are more likely to develop intracranial ischaemia, infarction foci, and even complicated situations with haemorrhage. This association is consistent with the intracranial haemorrhage foci found in this study, which showed that CMS patients had multiple cerebral ischaemia and infarct foci. Our previous study found that compared to that of healthy patients, the cerebral blood flow (CBF) of CMS patients is lower, and the average duration (MTT) is extended, indicating that the CMS patient brain tissue is in a hypoxic and ischaemic state. The number of erythrocytes in CMS patients showed a compensatory increase, and erythrocyte accumulation and haemoglobin levels were significantly elevated to increase the oxygen-carrying capacity of blood and relieve the body of hypoxia. The blood of CMS patients remains in a highly viscous state for a long time, which can cause organ damage. The main pathological manifestations of brain damage were vessel rupture or hyperaemia of the spinal meninges and skull base, intracerebral haemorrhage, swelling of the brain cells and interstitial oedema^12^. In this study, we also observed shallow sulci, fissions and narrowing of the ventricular system in CMS patients, which was consistent with previous studies.

The pathological changes after cerebral hypoxia mainly depend on the duration and degree of hypoxia. Under the condition of acute continuous hypoxia, the involved nerve tissue will undergo complete necrosis. In chronic hypoxia at high altitudes, the changes in brain tissue are mainly characteristic changes, and different neurons have different tolerances to hypoxia, even in the same hypoxic state. The loss and injury of different neurons are different. The neurons that are sensitive to hypoxia may be necrotic, while the more tolerant neurons may only have structural changes. Glial cells have a strong tolerance to hypoxia, so instead of dying, these cells may make proliferative changes in some regions^13^. The results of this study showed that the different brain tissues of CMS patients were affected differently by the hypoxic environment. In the CMS group, the FA value of the white matter of the right frontal lobe was lower than that of the control group, indicating that under a hypoxic environment, the microstructural damage of white matter in this area was more significant than that in other parts, and the consistency of white matter was low. In the CMS group, there was a positive correlation between FA in the left caudate nucleus and Hb and a negative correlation between FA in the left anterior limb of the caudate nucleus and cognitive function (MMSE). These results suggest that hypoxia may not cause nerve and fibroblast damage in the left caudate nucleus or the left internal capsule; on the contrary, hypoxia may cause regeneration in those regions. Some studies^14^ show that a small but statistically significant decrease in the apparent diffusion coefficient (ADC) was seen in some brain regions during acute hypoxia, whereas ADC was slightly elevated in high altitude as compared to sea-level adaptation. In this study, the ADC value of the right anterior limb of the internal capsule in the CMS group was higher than that of the control group, and the ADC value of the left thalamus of the CMS patients increased with increasing Hb, which is consistent with the pathological findings in CMS patients. Cognitive function is a variety of conscious mental activities, including simple perception, determination, understanding, judgement to the environment or to oneself, and the completion of complex mathematical calculations. Cognition is always present when human beings are conscious^15^, Studies have shown that CMS is an important reason for declined brain function^16^. Recent studies have shown that high-altitude migrants who work and live under low pressure and hypoxia can have damaged mobility and brain function to varying degrees. The intelligence of these high-altitude individuals is reduced; memory, especially short-term memory and transient memory, is significantly impaired, cognitive ability is reduced, and ability to react and judge is decreased^17^. Schlaepfer^18^ shows that continuous (>10 hours) stays at high altitude or 5000 metres above sea level will lead to damaged cognitive function. Wu *et al*.^19^ found that with increasing altitude, the presence of negative emotions increased accordingly, and emotional disturbances appeared in the environment of high-altitude hypoxia. Pagani^20^ suggested that appropriate adaption to the high-altitude environment can alleviate the cognitive abnormalities caused by hypoxia and even make these abnormalities disappear. In this work, we studied the relationships among the FA, ADC value, severity of the disease and cognitive function in patients with CMS and found that the ADC value of the right hippocampal area in CMS patients was negatively correlated with the MMSE score. The results indicate that the changes in the bilateral structures of the brain are different in CMS patients with chronic hypoxia, while the right hippocampal structure is correlated with changes in cognitive function.

In conclusion, CMS patients are prone to intracranial ischaemia, infarction and haemorrhage in the brain. Multiple structural changes occur in the brains of CMS patients compared to those of healthy subjects, and the damage has a correlation with the severity of the disease and cognitive function impairment. The white matter fibre bundles of CMS patients had no obvious damage except for the 3 patients with ischaemic foci.

## Methods

### Statement

All methods were carried out in accordance with relevant guidelines and regulations.

All experimental protocols were approved by the Research Ethics Committee of the Affiliated Hospital of Qinghai University.

We confirmed that all subjects provided informed consent.

### Study subjects

In total, 15 healthy volunteers and 17 CMS patients were enrolled. The CMS patient selection was based on “the criteria for the diagnosis of chronic mountain sickness in Qinghai” published by the sixth Academic Conference of Altitude Medicine and Hypoxic Physiology. The exclusion criteria are as follows: 1. chronic lung disease, emphysema, bronchiectasis, alveolar fibrosis, or lung cancer; 2. hypoxemia or chronic respiratory disorder caused by certain chronic diseases and resulting in secondary erythropoiesis; and 3. living at an altitude below 2500 m. All subjects signed the informed consent form before the examination.

### MRI scan

The MRI exam was performed on a Philips Achieva 3.0 T x-series superconducting scanner equipped with an 8-channel head coil. A routine brain scan was performed, followed by a DTI scan. The DTI sequence parameters are spin-echo Echo-planar Imaging (SE-EPI), repetition time (TR) = 6452 ms, echo time (TE) = 68 ms, field of view (FOV) 224 mm, b value = 0 or 1000 s/mm^2^, matrix 256 × 256, slice thickness 2 mm, slice distance 0 mm, total 60 slices, 16 diffusion directions, and a scan time of 4 min 5 s. Before the examination, all the subjects relaxed while lying on the examination bed, breathed calmly and closed their eyes without any thinking. The scans were acquired by a senior technician. First, routine a cranial MRI sequence was performed. A senior professional imaging diagnostician determined whether the following conditions were present for exclusion:1.obvious intracranial space-occupying diseases, such as a tumor; 2. large cephalic artefacts or did not complete the scan; 3. obvious breakage of the white matter fibre bundles found in the post-processing workstation; and 4. did not complete the MMSE score after the scan.

### Image processing and analysis

The Philips Extended MR Work Space 2.6.3.2 was used for image post-processing to reconstruct the ADC, FA and FA colour maps. Regions of interest (ROIs) were selected in the frontal white matter, caudate nucleus, anterior and posterior limbs of the inner capsule, external capsule, corpus callosum, thalamus, cingulate, central region of the semiovary and hippocampal gray matter and white matter. The same ROIs were selected for all study objects. ROIs in the CMS group included the areas with lesions. The FA and ADC values within the ROIs were measured. For all the subjects, the following fibre tracts were generated: the corticospinal tract, the cortical nucleus tract, the internal capsular colliculus system, the corpus callosum, the cingulate band, the superior longitudinal bundle, the lower longitudinal bundle, the superior occipital frontal tract, the inferior occipital frontal tract, the hook bundle, and the arcuate fibrous area. The morphology, number, shape, and distribution of the nerve fibre bundles in each selected area and the connections between various fibre bundle structures were analysed.

### Statistics

SPSS18.0 software was used to test the data for a normal distribution and the homogeneity of variance. The two samples were both normally distributed, and the total variances were the same. The two groups were tested by two independent samples t-tests, and the test level was ɑ = 0.05. In the CMS group, Pearson correlation analysis was performed for Hb, MMSE score, and FA and ADC values in each ROI.
